# New insights into trait introgression with the look-ahead intercrossing strategy

**DOI:** 10.1093/g3journal/jkad042

**Published:** 2023-02-23

**Authors:** Zheng Ni, Saba Moeinizade, Aaron Kusmec, Guiping Hu, Lizhi Wang, Patrick S Schnable

**Affiliations:** Department of Industrial and Manufacturing Systems Engineering, Iowa State University, Ames, IA 50011, USA; Department of Industrial and Manufacturing Systems Engineering, Iowa State University, Ames, IA 50011, USA; Department of Agronomy, Iowa State University, Ames, IA 50011, USA; Department of Industrial and Manufacturing Systems Engineering, Iowa State University, Ames, IA 50011, USA; Department of Industrial and Manufacturing Systems Engineering, Iowa State University, Ames, IA 50011, USA; Department of Agronomy, Iowa State University, Ames, IA 50011, USA

**Keywords:** trait introgression, intercrossing, backcrossing, look-ahead simulation

## Abstract

Trait introgression (TI) can be a time-consuming and costly task that typically requires multiple generations of backcrossing (BC). Usually, the aim is to introduce one or more alleles (e.g. QTLs) from a single donor into an elite recipient, both of which are fully inbred. This article studies the potential advantages of incorporating intercrossing (IC) into TI programs when compared with relying solely on the traditional BC framework. We simulate a TI breeding pipeline using 3 previously proposed selection strategies for the traditional BC scheme and 3 modified strategies that allow IC. Our proposed look-ahead intercrossing method (LAS-IC) combines look-ahead Monte Carlo simulations, intercrossing, and additional selection criteria to improve computational efficiency. We compared the efficiency of the 6 strategies across 5 levels of resource availability considering the generation when the major QTLs have been successfully introduced into the recipient and a desired background recovery rate reached. Simulations demonstrate that the inclusion of intercrossing in a TI program can substantially increase efficiency and the probability of success. The proposed LAS-IC provides the highest probability of success across the different scenarios using fewer resources compared with BC-only strategies.

## Introduction

Trait introgression (TI) is the process of introducing new traits from a donor line into an existing elite breeding line (i.e. the recurrent parent). Using this technique, breeders can produce a new line, which maintains most of the favorable alleles from the recurrent parent but also carries new alleles of interest from the donor. Typically, the donor carries favorable alleles of traits, which are absent from the recurrent parent. Compared with the donor, the recurrent parent performs better for the traits of economic interest, which is tracked by the presence of favorable background alleles.

A TI program consists of 2 alternating steps, selection and reproduction. In the selection step, breeders apply different selection strategies to choose promising crosses, which are made in the reproduction step. The offspring reflect the selection strategy mediated by recombination and Mendelian segregation. Because these latter two are not under breeders’ control, selection of parents and crosses are the main points at which breeders can influence TI. After an initial F1 cross between the donor and recurrent parents, repeated backcrossing (BC) to the recurrent parent is typically used to reduce linkage drag by eliminating undesirable alleles linked to the targeted, introgressed alleles (Lande and Thompson 1990; Hospital *et al.* 1992; Visscher *et al.* 1996; Khan *et al.* 2018). Hence, maximum introgression requires rare meiotic recombination events between the favorable allele from the donor and undesirable chromosomal segments from the donor parent. Once a sufficient level of introgression has been achieved, one or more generations of selfing are required to immortalize the new line (Ritland 1984; Jarne and Charlesworth 1993; Razanajatovo *et al.* 2016). Theoretical and simulation studies (Hillel *et al.* 1990; Lande and Thompson 1990; Gimelfarb and Lande 1994; Groen and Smith 1995; Frisch *et al.* 1999; Khan *et al.* 2018) and field experiments (Ragot *et al.* 1995) have shown that using marker-assisted BC strategies in TI programs is both time and cost-efficient. It allows the preservation of most of the favorable alleles from the recurrent parent while introducing the new alleles of interest at a predictable speed (Fisher 1949; Lande and Thompson 1990).

Linkage drag highlights the significant challenge posed to TI programs by the uncertainty of meiotic recombination events during reproduction. Visscher *et al.* (1996) proposed the background selection (BGS) based on the performance of the current generation. This improves the background recovery rate in the early generations of a TI program. However, because recombination rates vary across the genome, it is not enough simply to pick a parent with the target alleles and a high background recovery rate—the location of nonfavorable genomic segments from the donor parent is also important. Thus, a good breeding pipeline is required for a successful TI program (Frisch and Melchinger 2005). By making several strong assumptions, Han *et al.* (2017) used a genetic map to calculate a predicted cross value (PCV), the probability of obtaining a target gamete after 2 generations. The PCV method continues to increase the background recovery rate in later generations and is advantageous when the desired recovery rate is high. Moeinizade *et al.* (2021) proposed a look-ahead selection (LAS) method, which also used genetic maps to model recombination rates but used Monte Carlo simulations to account for longer program deadlines. All of these methods, however, are designed for the use of BC in TI programs.

An alternative to BC is intercrossing, mating 2 individuals from the backcross population to each other. This is usually used to create synthetic lines in multi-trait introgression (MTI) programs rather than the single-trait introgression (STI) programs described above (Soller and Plotkin-Hazan 1977). In MTI, multiple steps are needed (Peng *et al.* 2014a, 2014b; Sun and Mumm 2015). First, BC is used to produce some heterozygous candidates (Peng *et al.* 2014a). Then, intercrossing is used to combine the favorable alleles at all target loci into a single converted elite cultivar (QTL pyramiding) (Peng *et al.* 2014b). Then, a trait fixation step uses selfing to stabilize the target loci (Sun and Mumm 2015). Compared with MTI, STI only needs to focus on a single target locus. There is potential to combine the first 2 steps in MTI and perform them simultaneously.

The use of intercrossing, however, introduces potential complications. Unlike the recurrent parent used in BC, the individuals involved in intercrossing might have lower background performance. However, the combination of certain genotypes might provide more possibilities. In BC, only a group of individuals with at least one favorable allele at all target loci are eligible for selection because these alleles cannot be provided by the recurrent parents (Han *et al.* 2017; Moeinizade *et al.* 2021). Consequently, the candidate pool is more limited. Intercrossing solves this problem by providing more pairing candidates. More candidates, however, increase the computational load.

In this paper, we extend the BGS, PCV, and LAS methods to incorporate the potential for intercrossing in TI programs. Using data from a maize population, we conduct simulations to assess the efficiency of these 6 methods in introducing 3 target loci within 8 generations. We also discuss the strengths and weaknesses of the methods under different levels of resource availability and background recovery rate targets.

## Materials and methods

In this section, we will describe the problem by introducing the simulation pipeline and 3 previously proposed selection strategies for BC TI programs. After that, the proposed method, look-ahead intercrossing selection, will be presented. Here, we consider only diploid plants.

### Glossary of acronyms

**Table:** 

TL	The collection of target loci.
BL	The collection of background loci.
RC	The background recovery rate. $RC^{\text{goal}}$ is the goal background recovery rate.
BC	A TI breeding program applying only backcrossing.
IC	A TI breeding program applying both backcrossing and intercrossing.
GEBV	Genomic estimated breeding value.

### Nomenclature

**Table:** 

N	The total number of donor individuals.
P	The total number of markers for each individual.
M	The ploidy level of the organism (M=2 indicates diploid).
nTL	The number of target loci (major QTLs).
G	The binary marker matrix for N donors, $G\in \{0,1{\}}_{P\times M\times N}$, where $G_{\;p,m,i}$ indicates whether the pth allele on the mth chromosome of the ith individual is desirable (1) or not (0).
RF	The recombination frequency vector, $RF\in (0,0.5{]}_{(P-1)\times 1}$, where $RF_{p}$ is the recombination frequency between locus p and locus p+1.
W	The allele effect vector, $W\in [0,+\infty {)}_{P\times 1}$, where $W_{p}$ is the allele substitution effect for locus p.
VB	The background GEBV, $VB\in [0,+\infty {)}_{N\times 1}$, where $VB_{i}$ means the background GEBV of ith individual.
RCB	The background recovery rate, $RCB\in [0,1{]}_{N\times 1}$, where $RCB_{i}$ means the background recovery rate of ith individual.

### Problem definition

**Fig. 1.**
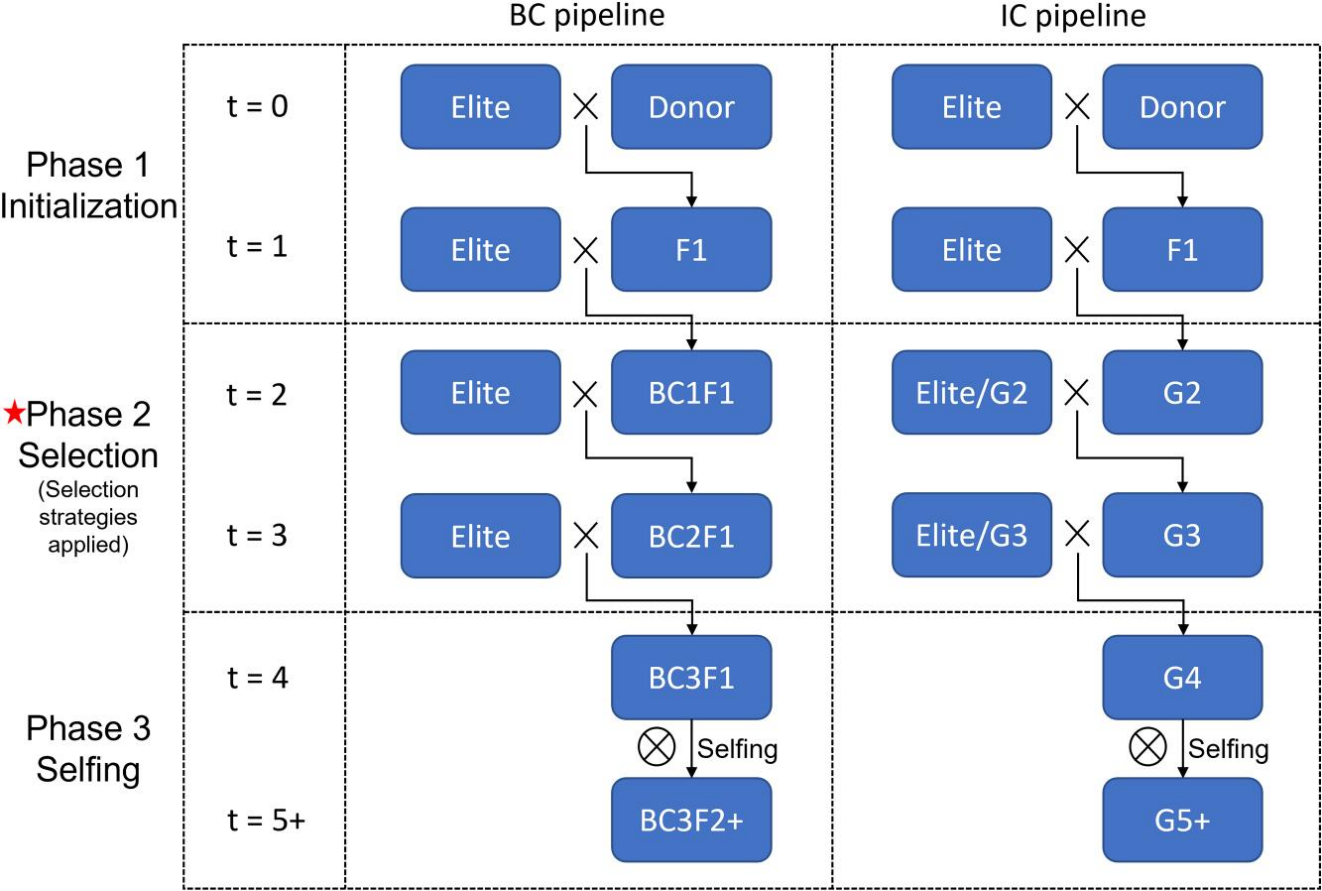
Two TI pipelines consisting of 2 generations of selection followed by repeated selfing. The left panel illustrates the traditional BC pipeline; the right, the proposed IC pipeline. The selection strategies described in the text are applied in Phase 2.

TI aims to transfer traits (the “target loci”) from a donor individual to the recurrent parent. Generally, the recurrent parent does not have favorable alleles at the target loci but is assumed to have favorable alleles at the remainder of the loci (the “background loci”). Meanwhile, the donor carries favorable alleles at the target loci but carries unfavorable alleles at the background loci. The donor and recurrent parents are usually completely inbred. The goal is to produce a new homozygous line that contains favorable alleles at the target loci and maintains most of the favorable alleles at the background loci within a specific number of generations of crossing. We will begin by introducing the general TI pipeline. A TI program can be split into 3 phases: the initialization phase, the selection phase, and the selfing phase (Fig. 1).

Before describing the general pipeline, we define 2 concepts.

Definition 1 The background recovery rate of the ith individual is

(1) $$RCB_{i}=\frac{\sum_{\;p\notin TL}\sum_{m=1}^{M}G_{\;p,m,i}}{(P-nTL)\cdot M},$$

where TL is the collection of target loci and nTL is the size of TL.

Definition 2 Given 2 individuals i and j and a set of loci, here we use the target loci TL whose size is nTL as an example, the genetic potential for these 2 individuals at these loci is

(2) $$GP_{i,j}=\frac{1}{nTL}\sum_{\;p\in TL}1\left(\sum_{m=1}^{M}(G_{\;p,m,i}+G_{\;p,m,j})\geq 1\right).$$

Here, function 1(⋅) is the indicator function.

Using these 2 concepts, we can label 2 groups of individuals, which will be used in the pipeline and selection strategies.

- *Positive individual*: We define a positive individual as one with at least one favorable allele at each of the target loci.
- *Successful individual*: We define a successful individual as one homozygous for the favorable allele at each target locus and with a background recovery rate greater than $RC^{\text{goal}}$. The generation with the first successful individual will be referred as the *successful generation*.

The initialization phase consists of 2 generations. The donor is crossed to the recurrent parent (t=0) to produce an F1 individual. Then, this F1 is backcrossed to the recurrent parent (t=1) to produce a segregating BC1F1 population from which selection begins.

In each generation of the selection phase, the best individuals are selected from the previous generation based on a selection strategy and a new generation is produced. In our pipeline, this procedure is performed twice (Fig. 1). The details of the selection strategies will be discussed later.

After the selection phase, repeated selfing is applied to increase homozygosity. We simulated generating 1,000 pollinated progeny from each self-pollinated individual in each generation, and the individual with the highest probability of producing successful individuals was selected. This selfing process is continued until a successful individual is produced. We refer to this generation as the successful generation and record it.

The 2 variants of this general pipeline that were simulated in this study are illustrated in Fig. 1. The left panel depicts the traditional and widely used BC pipeline (Ragot *et al.* 1995; Sun and Mumm 2015; Moeinizade *et al.* 2020). We refer to this pipeline as the BC pipeline. Each new backcross population after the F1 generation is only crossed with the recurrent parent during the selection phase. This ensures that at least 50% of the favorable background alleles are recovered during each generation. However, the ratio of favorable alleles at the target loci is at most 50% because the recurrent parent contains no favorable alleles at the target loci. To avoid losing these alleles, only positive individuals are considered candidates for selection. As the number of target loci increases, the pool of positive individuals will dramatically decrease. This is a significant drawback for the BC pipeline.

As an alternative, we propose a new breeding pipeline called the IC pipeline (Fig. 1, right). In this pipeline, we allow intercrossing during the selection phase. The new generation (Gt) can be crossed to both the recurrent parent (BC) and to the individuals in Gt itself (intercrossing). In this paper, we assume the absence of parent-specific effects: Reciprocal crosses are treated as identical. This allows the selection of individuals for crossing that may lack any favorable alleles at one or more target loci if the background recovery rate is high enough. This significantly enlarges the pool of candidate pairs and increases the chance of producing successful progeny.

### Selection strategies for the BC breeding pipeline

In this section, we outline 3 previously described selection strategies for TI programs based solely on BC. For these strategies, we assume the input individuals are positive individuals as we state in “Problem definition.”

#### Background backcrossing selection

Background backcrossing selection (BGS-BC) is a simple and efficient strategy that selects parents based on the background performance of the current generation. Commonly, the genomic estimated breeding value (GEBV) is used as the estimate of performance (Visscher *et al.* 1996; Meuwissen *et al.* 2001). The background GEBV (VB) for individual i is calculated as the weighted sum of the background genotype other than the target loci, using allele effects W as the weights:


(3) $$VB_{i}=\sum_{\;p\notin TL}\sum_{m=1}^{M}W_{p}\cdot G_{\;p,m,i}.$$

BGS-BC chooses the individuals with the highest background GEBVs as the individuals to cross with the recurrent parent. This strategy formalizes the intuition to choose the best candidates. However, this strategy focuses on the current generation’s performance and ignores linkage between loci and the breeding potential of the candidates.

#### Predicted cross value backcrossing selection

Predicted cross value backcrossing selection (PCV-BC) aims to choose pairs of individuals with a high probability of producing a gamete containing all favorable alleles after 2 generations, accounting for linkage and assuming random mating during the 2 generations (Han *et al.* 2017).

Let $g\in \{0,1{\}}_{P\times 1}$ denote the genotype of a random gamete produced by a random progeny from a proposed cross between 2 parents, P1 and P2. Then, the PCV of the selected parents is


(4) $$\mathrm{PCV}(P1,P2,RF)=\mathrm{Pr}(g_{p}=1,\;\forall \;p\in \{1,2,\ldots ,P\}).$$

Weuse the water pipe algorithm of (Han *et al.* 2017) to calculate PCV.

#### Look-ahead backcrossing selection

Look-ahead backcrossing (LAS-BC) selection is a Monte Carlo simulation method to estimate the probability of obtaining successful progeny in a target generation (Caflisch 1998; Bihani 2014; Moeinizade *et al.* 2021). The probability for each individual in the current generation is estimated by simulated BC to the recurrent parent. A random progeny from the simulated cross is selected for BC in subsequent generations. The final simulated generation consists of selfing to increase homozygosity. Multiple simulations are conducted for each individual to approximate the expected performance of the final population (Moeinizade *et al.* 2021). The individuals with the best-expected performance are selected to cross with the recurrent parent. Detailed procedures and analyses can be found in Moeinizade *et al.* (2021).

### Selection strategies for the IC breeding pipeline

In this section, we will introduce 3 intercrossing strategies that modify the 3 methods described above. The modifications enable the strategies to implement the IC pipeline (Fig. 1, right), which allows both BC and intercrossing.

#### Background intercrossing selection

Background intercrossing selection (BGS-IC) is similar to the original BGS-BC method, which is focused on the background information and the current generation performance. Given a group of individuals, $G\in \{0,1{\}}_{P\times M\times N}$, among which the first individual is the recurrent parent, we select individuals with the goal background recovery rate to create a candidate pool. Then, the BGS-IC strategy to select NC pairs can be formulated as the following integer linear programming (ILP) model:


(5) $$\max_{x}\;\sum_{i=1}^{N}\sum_{\;j=i}^{N}\left(\sum_{\;p\in TL}\sum_{m=1}^{M}W^{*}\cdot G_{\;p,m,i}+\sum_{\;p\notin TL}\sum_{m=1}^{M}W_{p}\cdot G_{\;p,m,j}\right)\cdot x_{i,j}$$

(6) $$\text{s.t.}\;\frac{\sum_{\;p\notin TL}\sum_{m=1}^{M}G_{\;p,m,i}}{(P-nTL)\cdot M}\geq RC^{\text{goal}}\cdot x_{i,j},\;\forall \;i,j\in \{1,\ldots ,N\}$$

(7) $$\;\sum_{m=1}^{M}(G_{\;p,m,i}+G_{\;p,m,j})\geq x_{i,j},\;\forall \;p\in TL,i,j\in \{1,\ldots ,N\}$$

(8) $$\;\sum_{i=1}^{N}\sum_{\;j=i}^{N}x_{i,j}=NC$$

(9) $$\;\sum_{i=2}^{N}x_{i,j}\leq 1,\;\forall \;j\in \{1,\ldots ,N\}$$

(10) $$\;\sum_{\;j=2}^{N}x_{i,j}\leq 1,\;\forall \;i\in \{1,\ldots ,N\}$$

(11) $$\;x_{i,j}\in \{0,1\},\;\forall \;i\leq j\in \{1,\ldots ,N\}.$$

Inthis ILP, decision variable $x_{i,j}$ indicates Whether ($x_{i,j}=1$) or not ($x_{i,j}=0$) individuals i and j are selected for crossing. The object function (5) is to maximize the total performance of 2 parents, one based on genetic value at the target loci and the other based on background GEBV. Here $W^{*}$ is a constant, which is larger than $2\sum_{\;p\notin TL}W_{p}$. Constraint (6) requires that the selected individual i achieves the background recovery rate goal. Constraint (7) requires that the selected individuals i and j cover every target locus with a favorable allele. Constraint (8) limits the number of selected crosses. Constraints (9) and (10) allow every individual to be selected in no more than one cross, except for individual i=1, which is reserved for the recurrent parent in every generation.

#### Predicted cross value intercrossing selection

For predicted cross value intercrossing (PCV-IC) selection, we apply the same PCV calculation as before. However, the addition of intercrossing increases the computational demands of the method because in a population of size N with a recurrent parent, the number of possible pairs is N2(intercross)+N(self)+N(backcross). To reduce the computational load, we calculate PCV only for pairs containing the recurrent parent and individuals with the top 20% GEBVs. The strategy chooses the pairs with the highest PCV scores as parents of the next generation.

#### Proposed look-ahead intercrossing selection strategy

Look-ahead intercrossing (LAS-IC) selection introduces the evaluation of intercrosses and selfs into the LAS-BC strategy. Given a population of size N and the recurrent parent, we have N2+2N pairs to evaluate in contrast to the N pairs evaluated by LAS-BC. The core idea in LAS-IC is to create a candidate pair generation step preceding performance evaluation by look-ahead simulations.

#### Candidate pair filter

The LAS-IC is a Monte Carlo method. Unlike the LAS-BC, which only considers backcrosses, the LAS-IC needs to consider massive numbers of combinations of parents, no matter whether we select the cross in the current generation or in each generation during look-ahead simulation. This is why we need to construct this candidate pair filter to reduce the computational burden. The purpose for this filter is to select the pairs with the greatest potential to generate new lines with high background performance that are homozygous for the target loci. There are 3 criteria:

1. The pair should have 100% genetic potential in the target loci.
2. The pair should have a genetic potential in the background loci larger than $RC^{\text{goal}}$.
3. The remaining pairs should be sorted by the summation of their background GEBVs. The top proportion of pairs are advanced to the performance evaluation step.

The filter creates a fixed number of candidate pairs, which will be used in our proposed strategy. In contrast to BGS-IC and PCV-IC, the same individual can be used in multiple crosses; however, reciprocal crosses are excluded.

#### Main steps of LAS-IC strategy

**Fig. 2.**
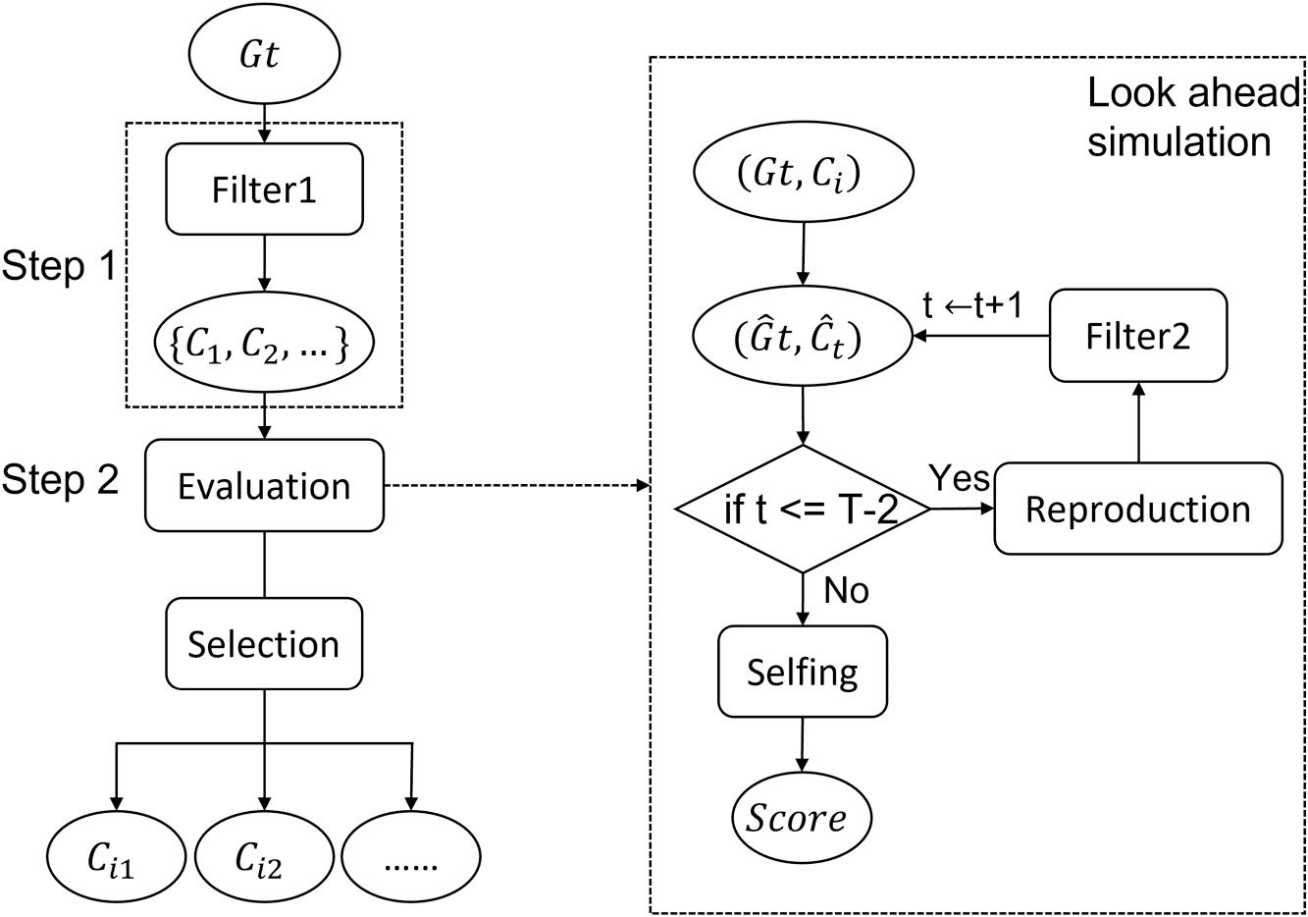
Structure of the LAS-IC selection strategy.

With the candidate filter introduced in section “Candidate pair filter,” the LAS-IC can be divided into 2 steps, candidate pair pool generation and performance evaluation. A brief structure is shown in Fig. 2. Detailed procedures are shown below:

*Step1 (Candidate pair pool generation):* This step selects candidate crosses for evaluation by simulations. The candidate pair filter described above is applied to the current generation Gt, and the top 0.1×N of the crosses are selected, where N is the population size of generation Gt. The candidate pair pool is denoted as $\left\{C_{1},C_{2},\ldots \right\}$.

*Step2 (Performance evaluation):* For each pair $C_{i}$ in $\left\{C_{1},C_{2},\ldots \right\}$ pool, we run look-ahead simulations to evaluate its performance.

In each simulated generation, we use a same candidate pair filter to produce a small pair pool. In the simulation, we use the top 0.05×N of the crosses, where N is the population size of generation Gt. The small-size filter helps us do the simulation faster. From the new pair pool, one random pair is selected to simulate the next generation. This is repeated until the selfing generation, T−1, is reached.

For the selfing generation, we pick the positive individuals with a size of $N_{\;pos}$ from the final generation, GT. We simulate the production of K progeny from each such individual by selfing. The maximum background GEBVs, VB, and the number of individuals homozygous for the favorable allele at all target loci and with the goal background recovery rate, $n_{i}$, are recorded. Then, the performance of cross $C_{i}$ in one simulation is


(12) $$V=\frac{1}{N_{\;pos}}\sum_{i=1}^{N_{\;pos}}\left(VB_{i}\cdot \frac{n_{i}}{K}\right),$$

Thesimulation is repeated 1,000 times and the expected performance of cross $C_{i}$ is the average of the V values.

Finally, the best-performing NC candidate pairs are selected to produce the next generation.

### Evaluation for selection strategies

For commercial breeding programs, the efficiency of the TI pipelines is highly important. Time and money are 2 main sources of cost (Lande and Thompson 1990; Gimelfarb and Lande 1994; Frisch *et al.* 1999). For some plant species, it may be possible to conduct one generation per calendar year; for others, multiple generations can be conducted per year. Considering the uncertainty of allele recombination, some projects are interested in the probability of success in specific generations.

**Fig. 3.**
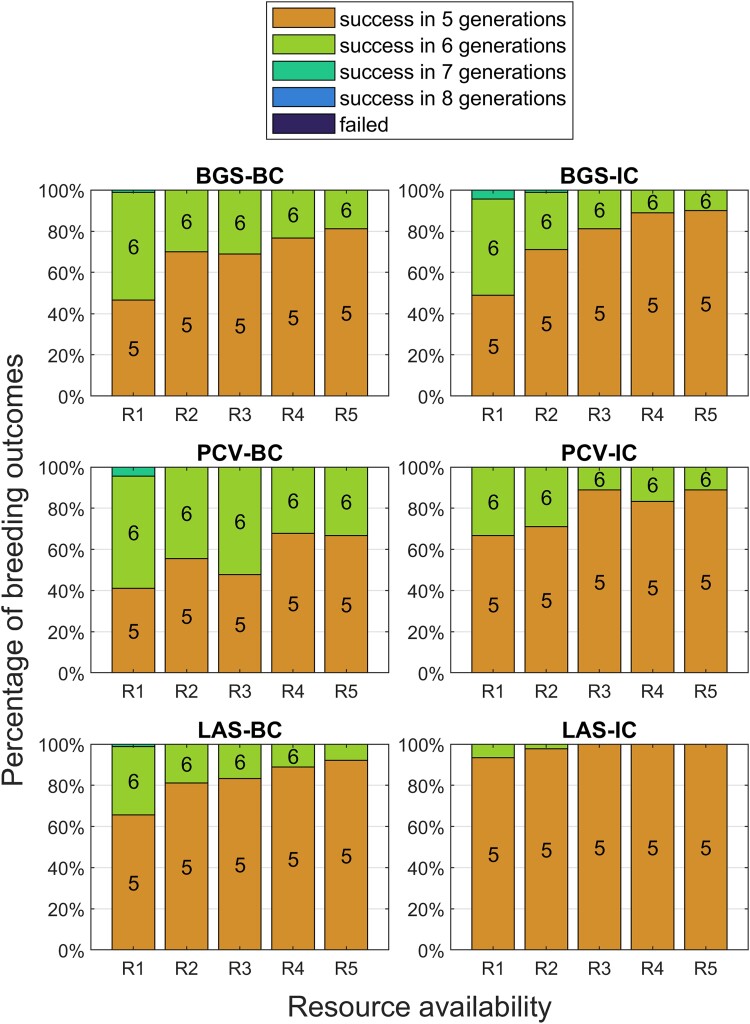
Outcomes of TI programs under different levels of resource availability using 90 donors with a goal background recovery rate of 0.96. The height of a bar represents the proportion of the TI programs that resulted in the indicated outcome; and the color of a bar indicates the outcome, which is either a success in a number of generations or a failure. For example, the “R1” column of bars in the BGS-BC subplot means about under the resource availability R1, about 45% of the TI programs succeeded in 5 generations, about 54% in 6 generations, and about 1% in 7 generations.

Considering all these factors, we apply a stacked bar plot, successful generation plot. Most of our results are based on this plot. One example is shown as Fig. 3. The figure describes the distribution of successful generations when the successful individual is present, applying the 6 mentioned strategies across different resource allocation plans. We conducted simulations for each donor as described in the “Problem definition.” Then, we collect the successful generations for all donors as one group. Then, each bar in the plot will use one group of data. By looking at each group of 5 bars, we can easily compare the performance of one specific selection strategy under different resource allocation plans. By comparing the specific bar from all 6 groups, we can evaluate the performances of the different selection strategies under the same resource scenario. Sometimes, we may even find one strategy better than the other when it uses fewer resources. Using these plots, breeders can choose the most suitable scenarios and strategies according to their own situations.

### Data

The maize data consists of a diversity panel of 383 inbred maize lines that were selected to study genetic and phenotypic diversity of their shoot apical meristems (SAM) (Leiboff *et al.* 2015). A set of 583,602 SNPs was assembled from multiple sources: GBS on inbreds used in the Genomes to Fields collaboration (AlKhalifah *et al.* 2018), GBS on the Ames diversity panel (Romay *et al.* 2013), RNA-seq on the SAM diversity panel (Leiboff *et al.* 2015), and tGBS (Ott *et al.* 2017) on a subset of the SAM diversity panel and 2 doubled haploid (DH) populations derived from that subset.

Raw sequencing reads were obtained from the NCBI SRA, aligned, and SNPs called following the protocol of Ott *et al.* (2017) for each data source. Duplicate samples within each file were combined using the consensus rules in Romay *et al.* (2013). Each file was self-imputed using Beagle v5.1 (Browning *et al.* 2018). The imputed records for the 383 inbred lines were merged, self-imputed again, and then filtered for SNPs having minor allele frequency (MAF) >0.05 and heterozygosity ≤0.05. This set of SNPs was imputed onto the DH populations. The 583,602 SNPs with $r_{dosage}^{2}\geq 0.5$ were retained for further analysis. These SNPs were phased by Beagle v5.1 (Browning and Browning 2007), SHAPEIT v2.9 (Delaneau *et al.* 2012), and EAGLE v2.4.1 (Loh *et al.* 2016) with consensus phases determined by the algorithm of Al Bkhetan *et al.* (2019).

Measurements of SAM volume from Leiboff *et al.* (2015) were used to derive an empirical distribution of SNP effect sizes. Genotypic best linear unbiased estimators (BLUE) of SAM volume were obtained by fitting the following model to raw data from Leiboff *et al.* (2015):


(13) $$y_{ij}=\mu +s_{ij}+g_{i}+r_{\;j}+\varepsilon_{ij},$$

where$y_{ij}$ is the calculated SAM volume of the ith genotype in the jth replication; μ is the population mean; $s_{ij}$ is an indicator for early or late stage of SAM development with respect to the development of the next leaf primordium; $g_{i}$ is the fixed effect of ith inbred line; $r_{\;j}$ is the random effect of replicate with distribution $N(0,\sigma_{r}^{2})$; and $\varepsilon_{ij}$ is the residual with distribution $N(0,R\sigma_{e}^{2})$, where R is a diagonal matrix allowing heterogeneity in the residual variance by replicate. Estimates of $\mu +g_{i}$ were used as input phenotypes for further analyses.

The phenotypes and genotypes were used to estimate SNP effects on SAM volume using the BayesB model (Meuwissen *et al.* 2001) implemented in GenSel v4.1 (Habier *et al.* 2011). The 3 SNPs with the largest positive effects on SAM volume, which are located on chromosomes 3, 5, and 8 (Supplementary Figure S1), were designated major QTL for introgression. Because each target locus is on a different chromosome, there is no physical linkage between them to consider. A subset of 390 SNPs was selected by uniformly selecting SNPs on each chromosome, where the number of SNPs selected was proportional to the length of the chromosome. A genetic map was estimated for these SNPs using the genetic map for the maize NAM population (Li *et al.* 2015) with linear interpolation of SNPs between genetic markers (Supplementary Figure S2). Recombination rates were estimated using Haldane’s mapping function.

The 90 inbred lines that were homozygous for the favorable allele at all 3 synthetic major QTL were used as donor lines. The recurrent parent was defined as a line possessing the favorable allele for larger SAM volume at all loci except the 3 major QTL.

### Simulation settings

Simulations were conducted in JULIA v1.6.1. The CPU was a 32-Core AMD EPYC 7502. It took about 2.5 h for LAS-BC and 15 h for LAS-IC to finish all simulation results for each goal background recovery rate (e.g. $RC_{\mathrm{goal}=0.96}$ ).

The 6 selection strategies were applied to the introgression of 3 alleles into the recurrent parent from 90 donor lines. Each method was applied to 2 generations of selection after the creation of the F1 and followed by a repeated selfing procedure as depicted in Fig. 1. We assumed that each cross produced 200 progeny (K=200). One thousand Monte Carlo simulations were conducted for both LAS-BC and LAS-IC. The final recovery rate was initially set at 0.96 ($RC^{\text{goal}}=0.96$). We expect a good TI program to successfully terminate in the fifth generation.

We also considered 5 different resource availability levels in our simulations (Table 2). Each level is described by the number of crosses made in each generation, and the levels are ordered by increasing resource availability. R1 is the closest to actual commercial breeding practices (Moeinizade *et al.* 2021).

**Table 2.** Five resource availability levels applied in the simulation from limited resource case to abundant resource case The numbers indicate the number of available crosses in each generation.

Generation	Resource availability				
	R1	R2	R3	R4	R5
t=1	1	1	1	1	1
t=2−8	2	3	4	5	6

## Results and discussion

This section will describe the data and show the simulation results of comparing the intercrossing strategy with the BC strategies.

### Simulation results

#### Performance comparison

As shown in Table 1, while the BC strategies consider only the BC, the IC strategies will consider BC, intercrossing, and selfing at the same time. Due to the limitation of the BC, the BC methods can only do selection in “positive individuals,” which is the only part they utilize the target loci alleles. While LAS-IC also utilizes the target loci to filter the candidates, the constraint is more relaxed because of the participation of the intercrossing and selfing, which allows the more potential candidate and in return increase the probability of producing progenies with better performance. Other than the differences in allowed cross types, BGS is a short-sighted strategy and evaluates the performance (background GEBV) of the current generation t. PCV prefers the perfect gametes produced by the next generation, which actually evaluate the next 2 generations t+2. However, the LAS always targets on a specific future generation T, which is assigned before the introgression. This allows LAS to have flexible evaluation strategies, from long-term to short-term selection. Meanwhile, the LAS methods use the Monte Carlo simulation to evaluate the future performance, which requires no additional genotyping cost compared with the traditional BGS and PCV method.

**Table 1.** Comparison between all 6 strategies. The cross types column describes the types of crossings that are considered (BC, backcrossing; IC, intercrossing; SF, selfing). The “Probability” columns, which summarize the results in Figure 3, show the mean and standard error of the probability of success in 5 generations under different levels of resource availability.

Strategy	Cross types			Probability of success in 5 generations ($RC^{\text{goal}}=0.96$)				
	BC	IC	SF	R1	R2	R3	R4	R5
BGS-BC	✓	×	×	46.67% (0.0529)	70.0% (0.0486)	68.89% (0.0491)	76.67% (0.0448)	81.11% (0.0415)
PCV-BC	✓	×	×	41.11% (0.0522)	55.56% (0.0527)	47.78% (0.0529)	67.78% (0.0495)	66.67% (0.05)
LAS-BC	✓	×	×	65.56% (0.0504)	81.11% (0.0415)	83.33% (0.0395)	88.89% (0.0333)	92.22% (0.0284)
BGS-IC	✓	✓	✓	48.89% (0.053)	71.11% (0.048)	81.11% (0.0415)	88.89% (0.0333)	90.0% (0.0318)
PCV-IC	✓	✓	✓	66.67% (0.05)	71.11% (0.048)	88.89% (0.0333)	83.33% (0.0395)	88.89% (0.0333)
LAS-IC	✓	✓	✓	93.33% (0.0264)	97.78% (0.0156)	100.0% (0.0)	100.0% (0.0)	100.0% (0.0)

We compared the performance of all 6 selection strategies at a standard background recovery rate of 0.96 (Results at Table 1 and Fig. 3).

As shown in Fig. 3, at the lowest resource level R1, BGS-BC, and PCV-BC completed TI in 5 generations for less than 50% of the donors, with some requiring 7 generations. When the amount of resources was increased, the percentage of success in 5 generations increased to 80% for BGS-BC and 65% for PCV-BC. PCV-BC did not demonstrate an improvement over BGS-BC at any resource level. LAS-BC demonstrated a 65–90% success rate in 5 generations across all resource levels.

After allowing intercrossing, the ratio of success in 5 generations generally increased for all methods compared with BC only. For BGS-IC, no effect was seen at the lowest resource levels. However, for R4 and R5, there was a 10% improvement from 80% to >90%. By contrast, PCV-IC demonstrated an increased ratio of success in 5 generations across resource levels compared with PCV-BC, making it competitive with BGS-IC. For LAS-IC, >90% of donors succeeded by generation 5, and the success rate increased to 100% with more resources. LAS-IC at the lowest resource level matched or exceeded the performance of every other method at the highest resource level, consuming ∼1/3 as many resources.

#### Intercrossing vs. backcrossing

**Fig. 4.**
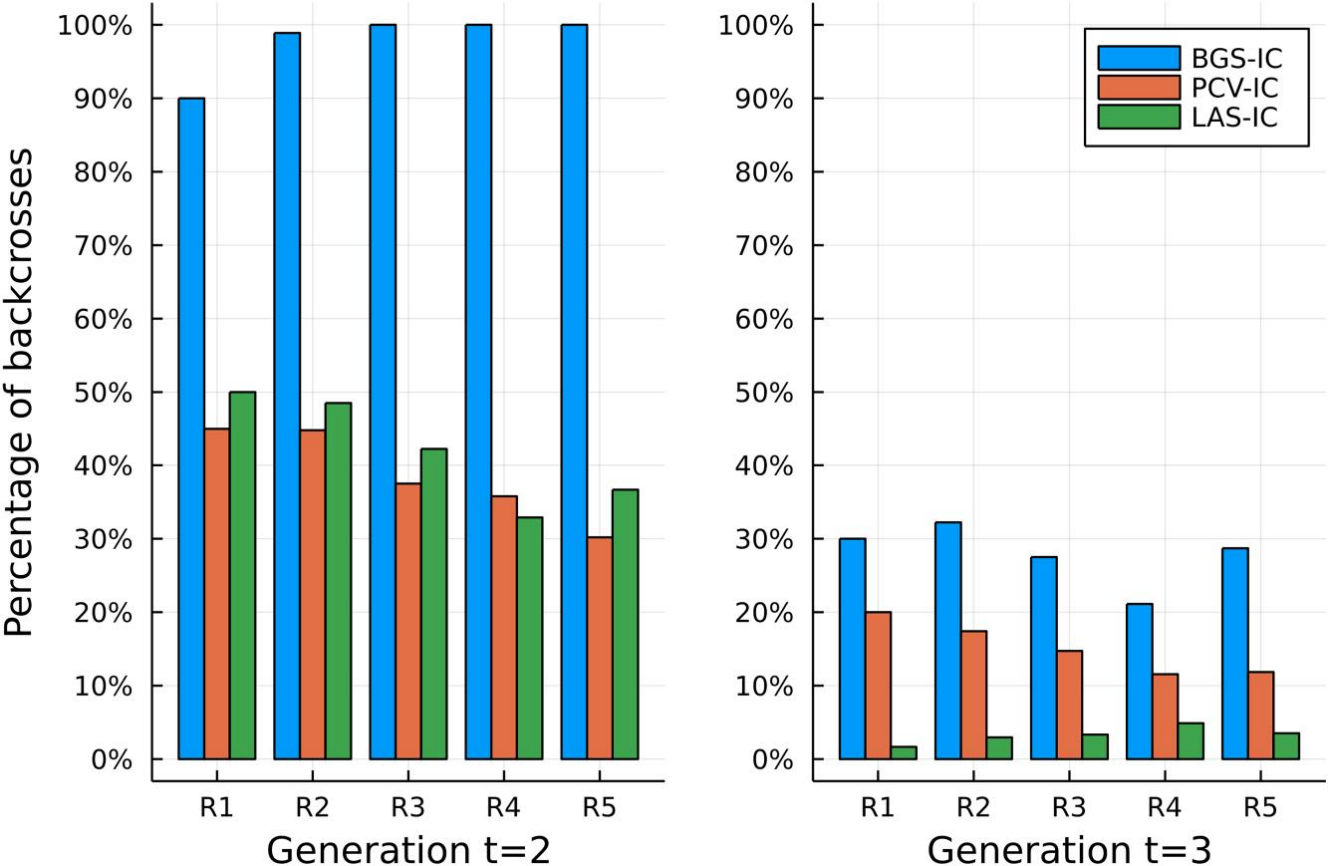
Percentage of backcrosses in generations t=2 and t=3 for 3 selection strategies that allow intercrossing.

While the BC methods can only select backcrosses, the intercrossing methods choose between the 2 crosses based on their evaluative criteria. Different intercrossing methods select different proportions of intercrosses based on generation and resource level (Fig. 4). The starkest differences are seen in Gen 2 where BGS-IC picks >90% backcrosses regardless of resource level, while PCV-IC and LAS-IC both select fewer than 50% backcrosses. In Gen 3, LAS-IC almost never picked BC pairs while other methods selected, on average 30% (BGS-IC) or 10% (PCV-IC) backcrosses. These patterns are consistent with the trends in comparative performance in Fig. 3 and an improvement in TI efficiency due to the incorporation of strategic intercrosses. Although selfing was not found to be an optimal cross in this simulation, this observation should not be generalized; under different scenarios with different timelines and resource availabilities, selfing could be an optimal cross.

#### Sensitivity to background recovery rate

A higher background recovery rate selects for lines with comparable performance to the original recurrent parent but is also expected to decrease the probability of obtaining the goal rate in earlier generations. We, therefore, compared results when the goal background recovery rate varied from 0.95 to 0.98.

**Fig. 5.**
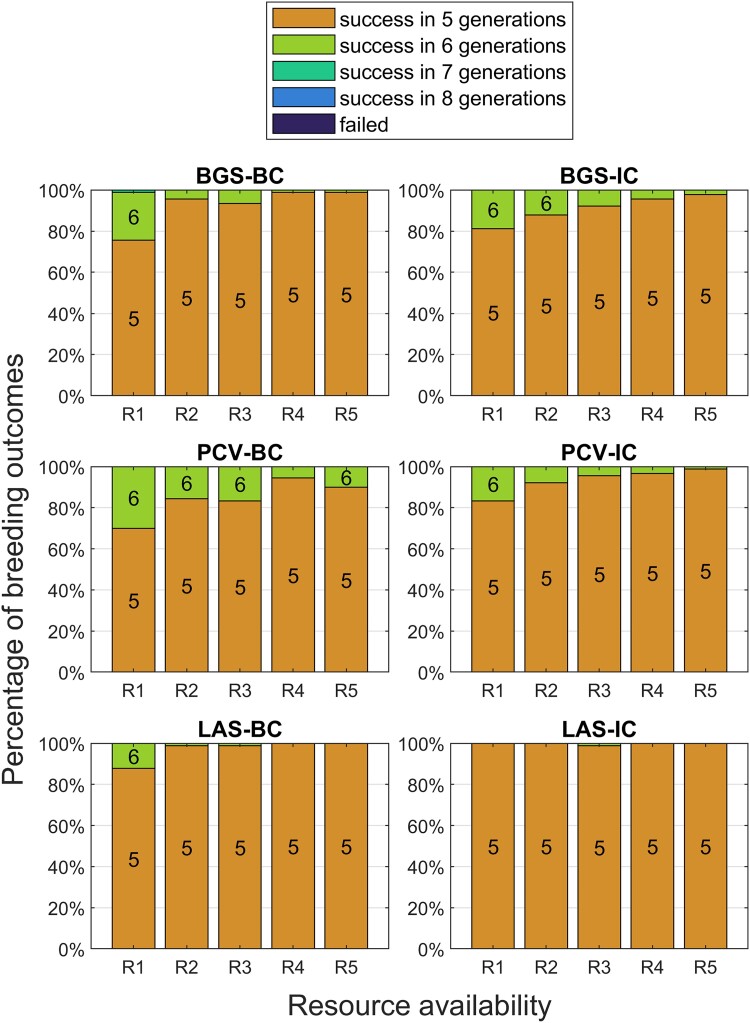
Outcomes of TI programs under different levels of resource availability using 90 donors with a goal background recovery rate of 0.95.

Figure 5 shows the results when $RC^{\text{goal}}=0.95$. For this easier goal, success rates are higher across all methods and resource levels compared with Fig. 3. The intercrossing methods demonstrate minor improvements over their backcross counterparts. LAS-IC practically ensures success in the fifth generation. At the lowest resource level, the 2 backcross methods’ performances improved a little, but only 75–85% of donors achieved success in 5 generations, which demonstrates the limitations of the BC strategy.

**Fig. 6.**
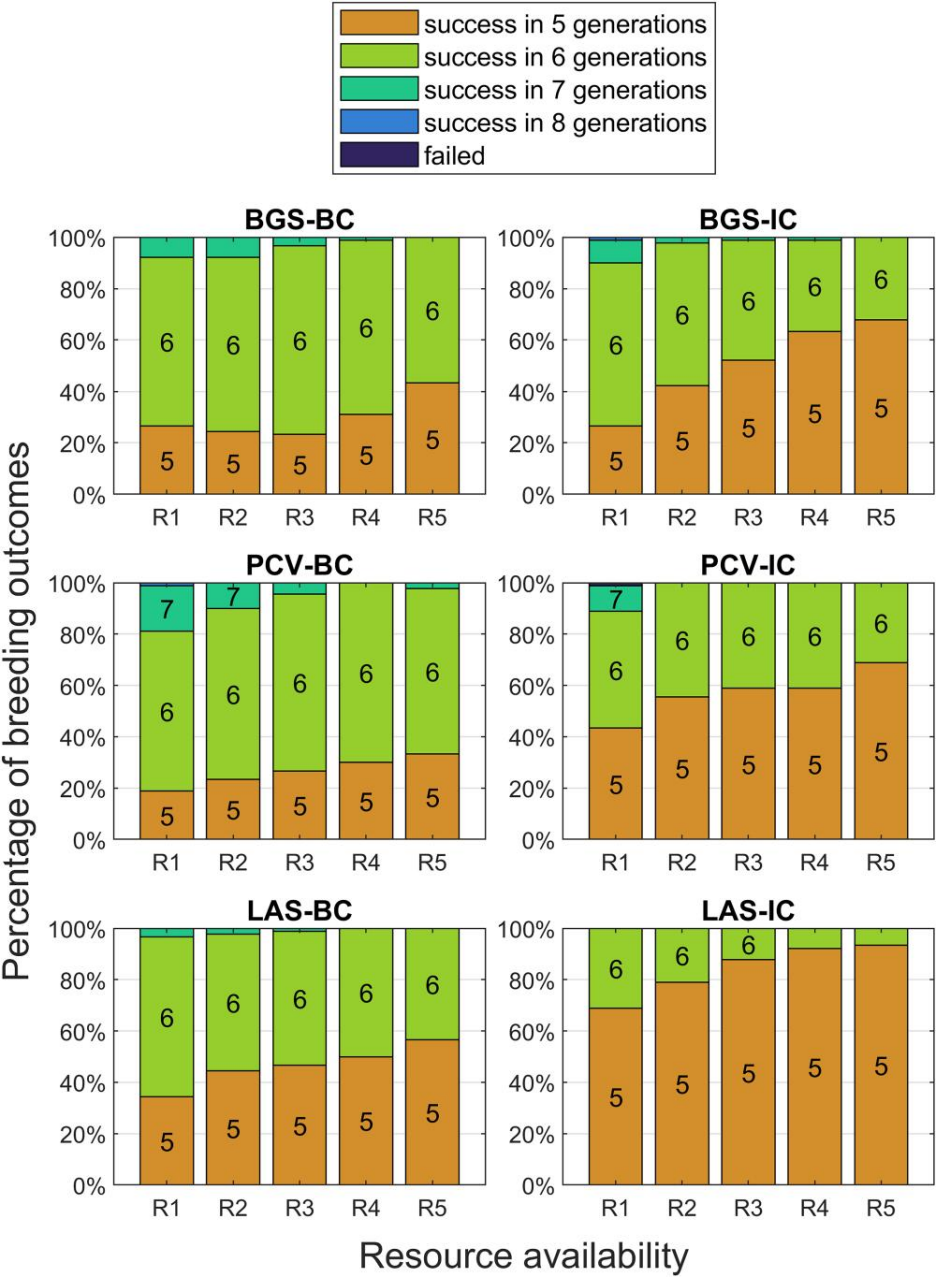
Outcomes of TI programs under different levels of resource availability using 90 donors with a goal background recovery rate of 0.97.

Increasing the goal to 0.97 revealed large differences between the 3 BC methods and their intercrossing variants. Figure 6 shows that 20–40% of donors were successful in 5 generations regardless of selection method for BC. Increasing resources had a minimal impact. The intercrossing methods generally outperformed their BC counterparts with increased resources providing a 20–30% improvement to BGS-IC and PCV-IC. LAS-IC, however, had a 70–90% success rate in 5 generations depending on resources.

**Fig. 7.**
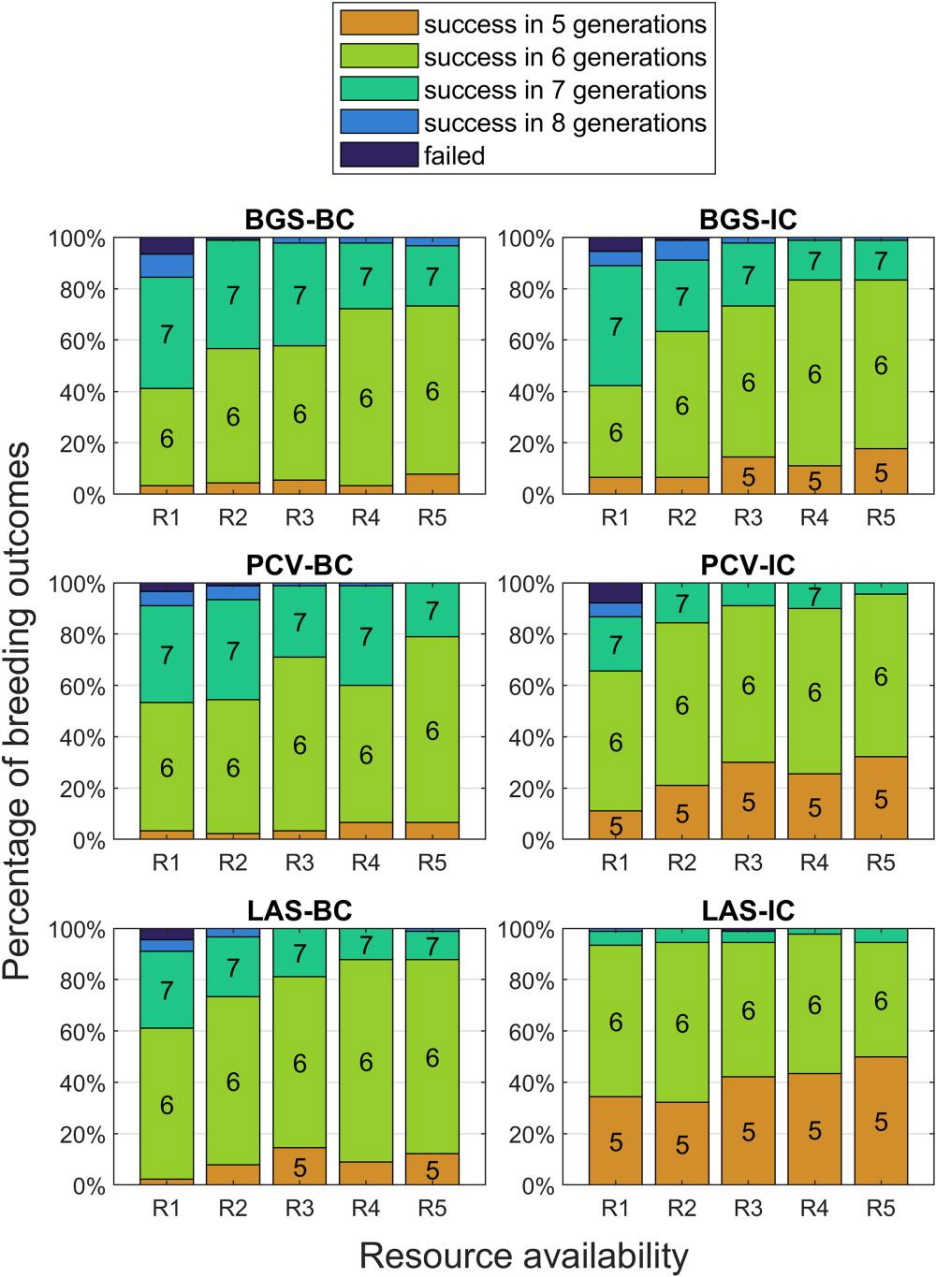
Outcomes of TI programs under different levels of resource availability using 90 donors with a goal background recovery rate of 0.98.

Finally, we increased the goal rate to 0.98 (Fig. 7). All 3 BC methods, BGS-IC, and PCV-IC had donors that failed outright at low resource levels, and success rates in 5 generations were generally <20%. By considering recombination rates in its calculations, PCV-IC outperformed BGS-IC by 10–15%. LAS-IC achieved success in the fifth generation for 30–50% of the donors and for >95% of donors by generation 6 without outright failure. This is a promising result but highlights the difficulty of such a stringent goal.

### Discussion

Advances in computational capacity have enabled more realistic and larger simulations of breeding programs. This enables researchers and plant breeders to assess the potential impacts of different breeding pipelines and selection strategies on target outcomes more rapidly and flexibly. In this study, we modified 3 previously proposed selection strategies for TI programs to allow for intercrossing between parental candidates in addition to the traditional backcross to the recurrent parent. These new methods increased the efficiency of the simulated TI programs relative to BC methods for different levels of resource availability. In particular, the LAS-IC method introduced intercrossing and additional selection criteria to focus computational resources on the most promising individuals. This enabled a success rate for LAS-IC at the lowest resource level equal to or greater than that of any other method at the highest resource level. These improvements also make feasible a more stringent background recovery rate.

While the improvements we have documented are encouraging, there are several avenues for further research and improvement.

First, we applied fixed resource allocation plans in our analyses. However, recent research on genomic selection programs has demonstrated that dynamic resource allocation in each generation can improve performance in the final generation by jointly considering selection, mating, and resource allocation decisions (Moeinizade *et al.* 2022). Such an approach may further improve the time and resource efficiency of LAS-IC.

Second, we consider the simultaneous introgression of 3 major QTLs from a single donor. However, more complex scenarios exist, including the introgression of more loci for one or more traits, the need to use multiple donors, or the improvement of multiple elite lines. Increasing the number of target loci makes it more likely that we would have to relax the requirement that a candidate cross possesses at least one favorable allele at each target locus, introduce a constraint to guard against the loss of the favorable allele at any of the target loci across the entire population, and relax the time constraints on the entire TI program. It also increases the probability that two or more target loci are located on the same chromosome arm. The primary impact of linkage between target loci would be to require one or more favorable recombination events between target loci, which would manifest mostly as an increase in the size of the candidate populations to improve the probability of observing such recombination events. Compared with existing strategies, LAS-IC considers more candidate crosses and the utilization of the recombination frequency may also provide great benefits for LAS-IC to deal with these scenarios.

Third, much research considers only diploid plants. However, commercial plants exhibit a range of ploidy levels, e.g. commercial strawberries are octoploid and seedless watermelons are triploid (a cross between diploid and tetraploid parents). These ploidy levels and crossing systems pose unique challenges that may require algorithmic refinements.

Finally, gene editing has the potential to speed up TI by directly introducing favorable alleles into elite cultivars. However, these techniques still require several generations for recovery of a successful transformation event and depend on knowledge of causal mutations, the transformability of elite cultivars, and differing regulatory regimes, among other factors. While these remain barriers to the deployment of gene editing technologies in TI, further research to optimize the traditional TI process is needed.

## Supplementary Material

jkad042_Supplementary_Data

## Data Availability

The datasets used in the computational experiments were derived from sources in the public domain as described in Section “Data.” Julia codes used to produce the results were uploaded to https://github.com/TroubleZN/Look-ahead-intercrossing-selection. Supplementary material are available at G3 online.
