# Supplementary material for: New insights into trait introgression with the look-ahead intercrossing strategy
Source: G3 (Bethesda). 2023 Feb 23;13(4):jkad042. doi: 10.1093/g3journal/jkad042 (PMC10085795; doi:10.1093/g3journal/jkad042)
Supplement: jkad042_Supplementary_Data [file jkad042_supplementary_data.pdf]

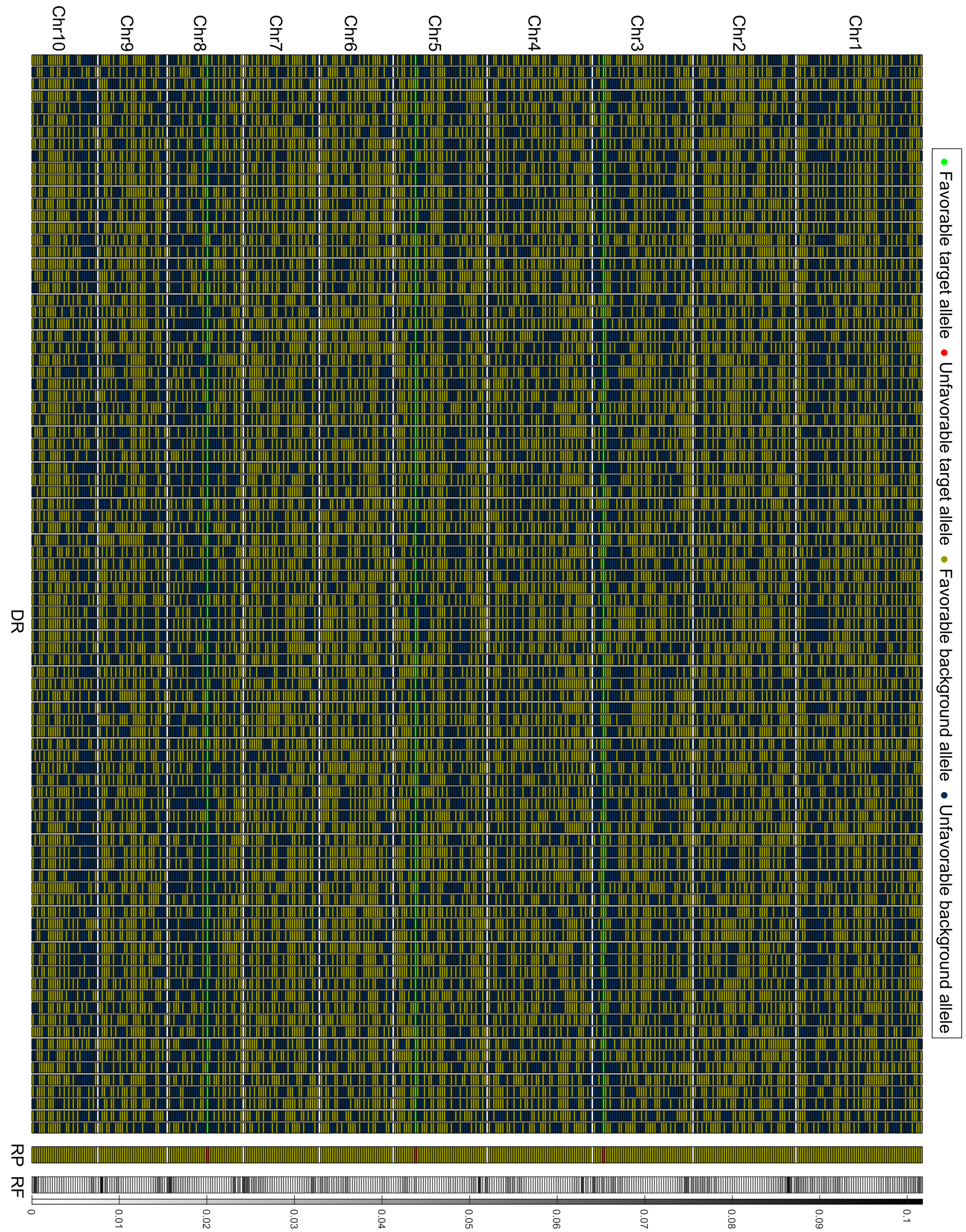

Figure S1: The genetic information of all the 90 donors and the recurrent 30 parents. DR are the donors' genetic information. Each bar in the genetic square is one donor. The blank blocks in the square are the breaks between the two chromosomes. The RP is the genotype of the recurrent parents. The RF is the heat plot for the recombination frequency. The segment between the chromosomes is set as 0.

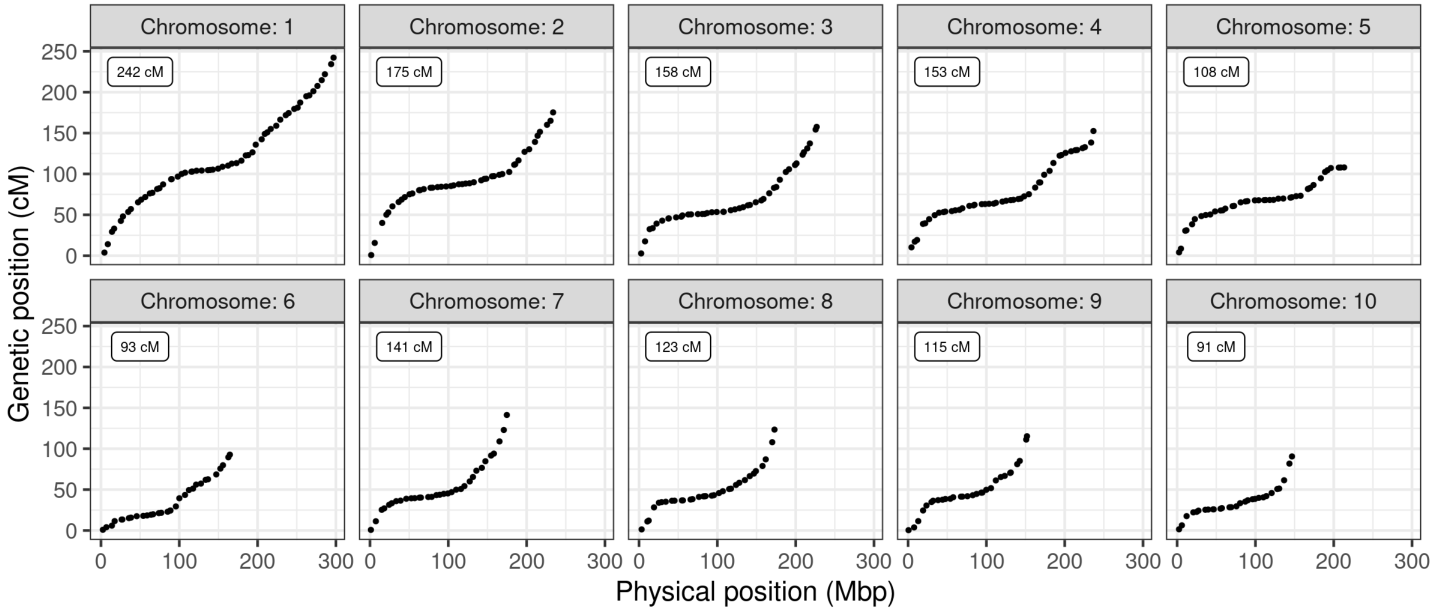

Figure S2: Physical (Mbp) and genetic (cM) positions of 390 markers distributed across the 10 maize chromosomes. The map length for each chromosome is provided in the insets. The total map length is 1,399 cM.
